# Combater o Sangramento – Um Apêndice de Cada Vez

**DOI:** 10.36660/abc.20220351

**Published:** 2022-07-07

**Authors:** Stefano Garzon, Willterson Carlos Bandeira

**Affiliations:** 1 Hospital Israelita Albert Einstein – Cardiologia Intervencionista São Paulo SP Brasil Hospital Israelita Albert Einstein – Cardiologia Intervencionista, São Paulo, SP – Brasil; 2 Irmandade da Santa Casa de Misericórdia de São Paulo - Cardiologia Intervencionista São Paulo SP Brasil Irmandade da Santa Casa de Misericórdia de São Paulo - Cardiologia Intervencionista, São Paulo, SP – Brasil

**Keywords:** Anticoagulantes/uso terapêutico, Hemorragia, Arritmias Cardíacas, Apêndice Atrial, Fibrilação Atrial, Acidente Vascular Cerebral/complicações

A fibrilação atrial (FA) é a arritmia cardíaca mais comum,^1^ afetando aproximadamente 80% da população com 80 anos ou mais.^2^ Aumenta o risco de acidente vascular cerebral cardioembólico em 5 vezes em todas as idades^3^ e está relacionado a mais de 20% dos acidentes vasculares cerebrais em pacientes acima de 80 anos. Os AVCs embólicos costumam ser mais graves do que outros AVCs,^4^ e os anticoagulantes são a base do tratamento, fundamentais para reduzir o risco cardioembólico nessa população. No entanto, a decisão de iniciar anticoagulantes orais nem sempre é simples e requer avaliação dos riscos embólicos e hemorrágicos.^5^ O risco embólico em pacientes com FA geralmente é avaliado usando sistemas de pontuação padronizados, como o escore CHA2DS2-VASc,^6^ mas pode ser refinado usando outros dados clínicos, como tamanho do átrio esquerdo^7^ e a duração da FA.^8^ O risco de sangramento geralmente é avaliado pelo escore HAS-BLED,^9^ sendo os sangramentos graves mais comuns em pacientes mais velhos.^10^ Por essa razão, os médicos muitas vezes têm medo de iniciar a anticoagulação em pacientes mais velhos, embora as evidências atuais mostrem que geralmente é seguro usar anticoagulantes orais na maioria desses pacientes.^11^ No entanto, sangramentos maiores podem ocorrer em até 3% dos pacientes em uso de anticoagulantes orais,^12^ sendo necessária a interrupção do tratamento.

Mais de 90% de todos os trombos do átrio esquerdo se originam no AAE,^13^ e a redução do risco com oclusão do AAE é comparável à anticoagulação.^14^ Por esse motivo, a oclusão percutânea do AAE surgiu como tratamento alternativo para pacientes com contraindicação à anticoagulação oral ou evento embólico em uso de anticoagulantes orais. Há evidências crescentes de que a oclusão do AAE é segura e viável na maioria dos pacientes,^15–17^ e essa experiência inicial multicêntrica de oclusão do AAE com o dispositivo baseado

em plug LAmbre no Brasil^18^ mostra resultados semelhantes à literatura médica atual. Neste estudo, 74,6% de todos os pacientes tiveram um episódio de sangramento maior usando anticoagulantes orais ou um acidente vascular cerebral apesar da anticoagulação oral. Os pacientes apresentavam alto risco embólico e de sangramento, com pontuação média de CHA2DS2-VASc de 4,6±1,7 e uma pontuação média de HAS-BLED de 3,4±1,1. A taxa de sucesso do procedimento foi de 100%, sem mortes ou acidentes vasculares cerebrais em um seguimento médio de 18±12 meses.

Nos Estados Unidos, nos primeiros três anos do NCDR Left Atrial Appendage Occlusion Registry,^17^ 38.158 pacientes foram submetidos à oclusão do AAE. Independentemente das diferenças regionais, parece haver uma diferença marcante com o Brasil. Naturalmente, o presente artigo^18^ não abrange a totalidade dos casos realizados no país, mas reuniu casos de 18 centros de todo o Brasil, sendo 51 casos realizados em 2 anos e meio. Os brasileiros estão envelhecendo e ficando mais frágeis,^19^ assim como seus pares em outros lugares. É razoável supor que a idade avançada e o aumento da fragilidade também aumentam os riscos de FA e sangramento nessa população. A questão que fica por responder é: por que a oclusão do AAE é tão raramente realizada no Brasil? É o custo? Ou há uma falta de consciência e, portanto, menos indicações? Onde devemos atuar para oferecer um melhor atendimento a esses pacientes?

Em conclusão, a oclusão percutânea do AAE é uma tecnologia comprovada. É uma alternativa segura, viável e eficaz aos anticoagulantes orais em pacientes com FA e com alto risco de eventos embólicos e hemorrágicos. Esperamos que o presente estudo^18^ ajude a divulgar um procedimento que não é comum no Brasil, ao contrário dos pacientes que provavelmente se beneficiarão dele.
