# Supplementary material for: Combater o Sangramento – Um Apêndice de Cada Vez
Source: Arq Bras Cardiol. 2022 Jul 7;119(1):57–8. [Article in Portuguese] doi: 10.36660/abc.20220351 (PMC9352134; doi:10.36660/abc.20220351)
Supplement: Translation

# Translation: Tackling Bleeding – One Appendage at a Time

**Authors:** Stefano Garzon, Willterson Carlos Bandeira

**Affiliations:** 1 São Paulo SP Brazil Hospital Israelita Albert Einstein – Cardiologia Intervencionista, São Paulo, SP – Brazil; 2 São Paulo SP Brazil Irmandade da Santa Casa de Misericórdia de São Paulo - Cardiologia Intervencionista, São Paulo, SP – Brazil

**Keywords:** Anticoagulants/therapeutic use, Hemorrhage, Arrhythmias, Cardiac, Atrial Appendage, Stroke, Atrial Fibrillation

Atrial fibrillation (AF) is the most common cardiac arrhythmia,^1^ affecting approximately 80% of the population aged 80 years or older.^2^ It increases the risk of cardioembolic stroke 5-fold across all ages^3^ and is related to more than 20% of strokes in patients above 80 years. Embolic strokes are often more severe than other strokes,^4^ and anticoagulants are the cornerstone of the treatment, paramount to reducing cardioembolic risk in this population. However, the decision to start oral anticoagulants is not always straightforward and requires assessing both embolic and bleeding risks.^5^ Embolic risk in patients with AF is usually assessed using standardized scoring systems such as the CHA_2_DS_2_-VASc score,^6^ but it can be further refined using other clinical data such as left atrial size^7^ and duration of AF.^8^ Bleeding risk is usually assessed using the HAS-BLED score,^9^ with severe bleedings being more common in older patients.^10^ For that reason, physicians are often fearful of starting anticoagulation in older patients, even though current evidence shows that it is usually safe to use oral anticoagulants in most of these patients.^11^ However, major bleeding can occur in up to 3% of patients using oral anticoagulants,^12^ requiring the interruption of the treatment.

Over 90% of all left atrial thrombi originate in the LAA,^13^ and the risk reduction with LAA occlusion is comparable to anticoagulation.^14^ For this reason, percutaneous LAA occlusion has emerged as an alternative treatment for patients with either contra-indications to oral anticoagulation or an embolic event while using oral anticoagulants. There is growing evidence that LAA occlusion is safe and feasible in most patients,^15–17^ and this initial multi-centric experience of LAA occlusion using the plug-based device LAmbre in Brazil^18^ shows similar results to current medical literature. In this study, 74.6% of all patients have had either a major bleeding episode using oral anticoagulants or a stroke despite oral anticoagulation. Patients were at high embolic and bleeding risks, with a mean CHA_2_DS_2_-VASc score of 4.6 ± 1.7 and a mean HAS-BLED score of 3.4 ± 1.1. The procedure's success rate was 100%, with no deaths or strokes at a mean follow-up of 18 ± 12 months.

In the United States, in the first three years of the NCDR Left Atrial Appendage Occlusion Registry,^17^ 38,158 patients underwent LAA occlusion. Regardless of regional differences, there seems to be a striking difference with Brazil. Naturally, the present article^18^ does not encompass the entirety of cases performed in the country, but it gathered cases from 18 centers across Brazil, with 51 cases being performed in 2 and a half years. Brazilians are aging and getting frailer,^19^ similar to their counterparts elsewhere. It is reasonable to assume that older age and increasing frailty also increase AF and bleeding risks in this population. The question that remains to be answered is: why LAA occlusion is so rarely performed in Brazil? Is it the cost? Or is there a lack of awareness and, therefore, fewer indications? Where should we act to deliver better care to these patients?

In conclusion, percutaneous LAA occlusion is a proven technology. It is a safe, feasible and effective alternative to oral anticoagulants in patients with AF and at high risk of both embolic and bleeding events. Hopefully, the present study^18^ will help spread the word about a procedure that is not common in Brazil, unlike the patients who are likely to benefit from it.
